# Involvement of the V2 Vasopressin Receptor in Adaptation to Limited Water Supply

**DOI:** 10.1371/journal.pone.0005573

**Published:** 2009-05-18

**Authors:** Iris Böselt, Holger Römpler, Thomas Hermsdorf, Doreen Thor, Wibke Busch, Angela Schulz, Torsten Schöneberg

**Affiliations:** 1 Institute of Biochemistry, Molecular Biochemistry, Medical Faculty, University of Leipzig, Leipzig, Germany; 2 Rudolf-Böhm-Institute of Pharmacology and Toxicology, Medical Faculty, University of Leipzig, Leipzig, Germany; 3 Department of Organismic and Evolutionary Biology and the Museum of Comparative Zoology, Harvard University, Cambridge, Massachusetts, United States of America; The Rockefeller University, United States of America

## Abstract

Mammals adapted to a great variety of habitats with different accessibility to water. In addition to changes in kidney morphology, e.g. the length of the loops of Henle, several hormone systems are involved in adaptation to limited water supply, among them the renal-neurohypophysial vasopressin/vasopressin receptor system. Comparison of over 80 mammalian V2 vasopressin receptor (V2R) orthologs revealed high structural and functional conservation of this key component involved in renal water reabsorption. Although many mammalian species have unlimited access to water there is no evidence for complete loss of V2R function indicating an essential role of V2R activity for survival even of those species. In contrast, several marsupial V2R orthologs show a significant increase in basal receptor activity. An increased vasopressin-independent V2R activity can be interpreted as a shift in the set point of the renal-neurohypophysial hormone circuit to realize sufficient water reabsorption already at low hormone levels. As found in other desert mammals arid-adapted marsupials show high urine osmolalities. The gain of basal V2R function in several marsupials may contribute to the increased urine concentration abilities and, therefore, provide an advantage to maintain water and electrolyte homeostasis under limited water supply conditions.

## Introduction

Over a period of 170 million years of mammalian evolution species adapted to a great variety of habitats with different accessibility to water. Several mammals managed adaptation to conditions of water restriction among them desert rodents and elephants. Interestingly, elephants still present anatomical and physiological features of their evolutionary origin as aquatic animals [1], [2]. Other closely related mammals such as Baikal and California seals managed adaptation to fresh and sea water conditions, respectively. There is strong evidence that, besides morphological modifications in the kidney [3], [4], several hormone systems [5], among them the renal-neurohypophysial hormone system of vasopressin, are involved in adaptation to differences in fresh water supply [6].

The peptides of the vasotocin/vasopressin/oxytocin family are evolutionary old hormones which are already found in agnate bony and cartilaginous fishes [7]–[9]. At least one homolog each of vasopressin and oxytocin (mesotocin, isotocin) is found in jawed vertebrates whereas jawless vertebrates possess vasotocin [10]. Along with the divergence of the vasopressin/oxytocin peptides their receptors developed co-evolutionary and in mammals at least two of the various vasopressin receptor subtypes and one oxytocin receptor became established within all the genomes investigated so far. All members of the vasopressin receptor family belong to the superfamily of rhodopsin-like G protein-coupled receptors (GPCR). In mammals V1 vasopressin receptors (V1aR, V1bR) are involved in blood pressure regulation and central feedback mechanism whereas the V2 vasopressin receptor (V2R) maintains the water balance and electrolyte homeostasis.

The V2R is mainly expressed in kidney and regulates aquaporin-2-mediated water reabsorption via activation of the G_s_ protein/adenylyl cyclases/cAMP pathway. Vasopressin receptor function specialized in the regulation of water and electrolyte homeostasis is obviously linked to the evolution of tetrapods [11] but there is evidence that vasopressin-regulated cAMP formation is already present in fishes [12].

The importance of the vasopressin/V2R system in the hypothalamic-renal regulation of the water and electrolyte homeostasis becomes evident when one of the components is inactivated. For example, inactivating mutations of the V2R gene cause an inherited form of nephrogenic diabetes insipidus (NDI) [13]. NDI is characterized by the inability to concentrate the urine and an increased urine volume. Inherited NDI is not only found in humans but also in mice, dogs and horses [14]–[16] indicating similar functional relevance of the V2R among mammals. However, there are several examples where patients managed NDI basically by appropriate water supply (family 4 in [17] and family 1 in [18]). This may suggest that partial or even full loss of V2R function is tolerated in species with free access to fresh water.

An opposite phenotype, the nephrogenic syndrome of inappropriate antidiuresis (NSIAD), is seen when V2R is altered by activating mutations. Here, the phenotype is characterized by serum hypoosmolarity and high urinary sodium levels [19]. This may suggest that increased activity of V2R provides some advantage in concentrating the urine in order to save water under dry environmental conditions. One can speculate that the limited alimentary water supply prevents serum hypoosmolarity in desert animals, a sign found in patients with NSIAD under water-*ad-libitum* conditions.

According to allosteric ternary complex models of receptor activation [20], V2R, like all GPCR, exists in an equilibrium between inactive and active conformations. The models predict that, even in the absence of agonist, a certain fraction of receptors will spontaneously adopt an active conformation, permitting agonist-independent G-protein activation. Agonist binding to the free receptor leads to stabilization of the activated form of the receptor that is able to couple to the G protein. Inverse agonists decrease the spontaneous activity of receptors by shifting the equilibrium towards the inactive stage. Like agonists or inverse agonists, activating or inactivating mutations are capable of shifting the equilibrium toward active and inactive conformations, respectively. The fact that structural variations of the V2R may have an impact on basal activity, efficacy and potency gives rise to a spectrum of adaptive functionalities.

Here, we set out to analyze whether structural and functional properties of mammalian V2R correlate to different environmental conditions. We focused on V2R rather than on the hormone vasopressin because it is also involved in blood pressure regulation and the latter physiological function may restrict the adaptive variability of the peptide hormone. Thus, 87 V2R ortholog sequences were retrieved from mammalian species, some existing in extreme habitats. This sequence information enabled us to identify motifs and residues that are important for maintaining the receptor function. Our functional studies provide evidence that increased basal V2R activity is probably responsible for increased basal urine concentration ability in some desert-living marsupials.

## Results and Discussion

Public sequence databases were mined for mammalian genomic V2R sequences. We further increased the sequence information by fully or partially cloning of additional V2R orthologs (see Supporting Material Table S1) with special focus on mammalian species living in desert environments and habitats with water excess. The sequence information of 87 full-length or partially identified V2R orthologs enabled us to determine and to compare structural parameters of the V2R gene and protein which are relevant for maintaining receptor's function.

### Preservation of the genomic structure of the V2R in mammals

Previous studies have shown that some structural variability of V2R is generated by receptor splice variants e.g. in rodents [21]–[23]. Further, mutations of splice sites within the V2R gene (*AVPR2*) were found to be the cause of inherited NDI [24]–[27]. Therefore, we analyzed the genomic structure of mammalian V2R genes in respect to the conservation of splice donor and acceptor sites.

**Figure 1:**
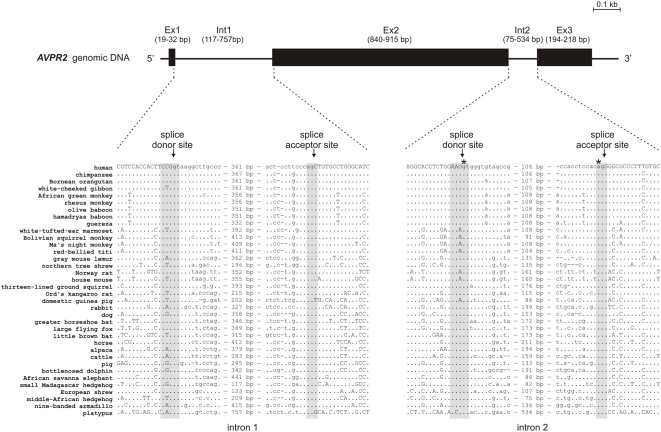
Genomic structure of mammalian V2R and evolutionary conservation of intron/exon boundaries. To determine the conservation of the genomic structure, the sequence of 87 mammalian V2R genes were retrieved by cloning from genomic DNA and by mining public databases. During 170 million years of mammalian evolution the genomic structure of three coding exons was preserved. There is experimental evidence that the V2R gene of several mammals including the human V2R gene contains an additional non-coding exon in 5′ position (not shown). Sequence alignments of the exon/intron boundaries indicate high conservation of the splice donor and acceptor sites. The positions of NDI-causing splice site mutations are marked with an asterisk.

Three exons encode for the human V2R [28] and this genomic structure appears to be evolutionary preserved among mammals (see Figure 1). The second intron is ancient and is found not only in V2R genes of birds and mammals but also in other members of the vasopressin/oxytocin receptor family at the same position [29], [30]. The first intron was probably introduced in the very early mammalian evolution because it is found in platypus, marsupial and other mammalian but not in avian V2R genes.

Although the intron sizes vary between 117 bp and 757 bp for intron 1 and 75 bp and 534 bp for intron 2, the core sequences of the donor and acceptor sites in mammalian V2R genes are highly conserved (see Figure 1). All three mutations in the human V2R proposed to affect pre-mRNA splicing [31] are at splice site positions that are 100% conserved during mammalian evolution.

### High structural conservation of V2R in mammals

**Figure 2:**
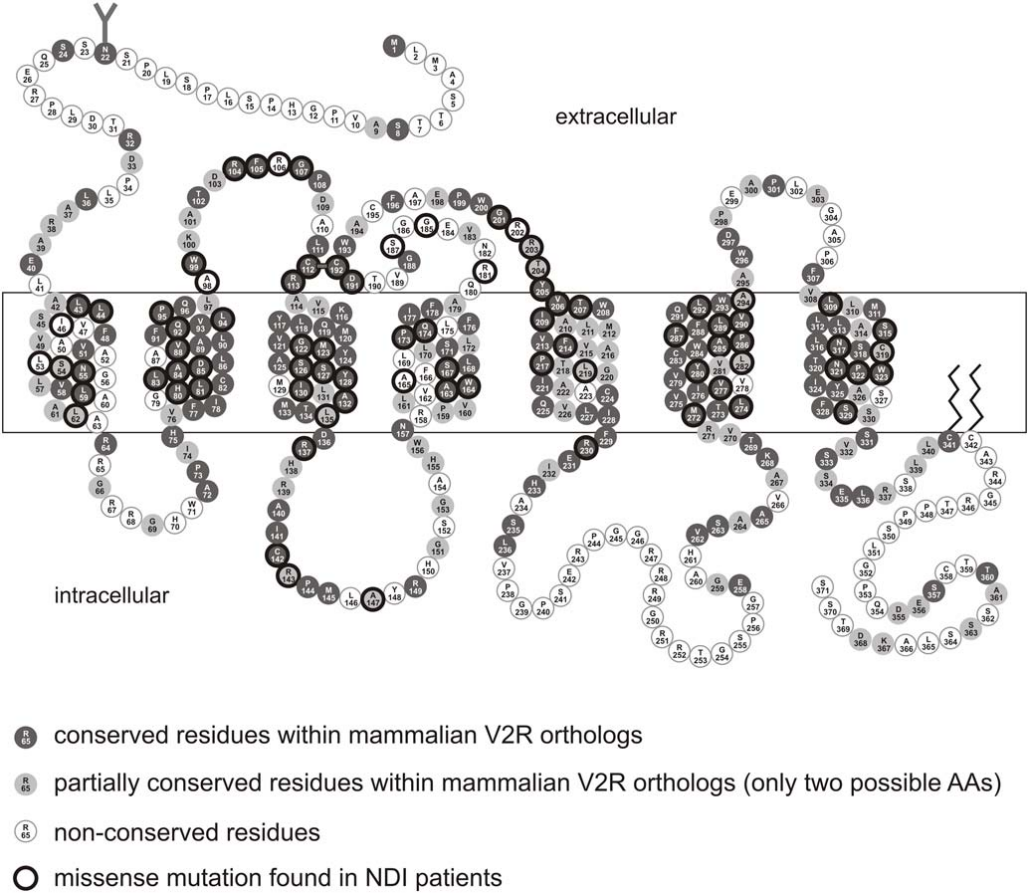
Structural conservation of the mammalian V2R. The amino acid sequence of the human V2R is shown. Positions conserved in mammalian V2R orthologs are depicted in dark grey. Positions which vary only by two amino acids are shown in light gray. Positions given in white are not preserved in mammals. For the N-terminal part (position 1 to 64, numbering refers to the human V2R amino acid sequence) we analyzed 38 ortholog sequences, for the middle part (position 65 to 322) 87 sequences and for the C-terminal part (position 323–371) 42 sequences. Over 103 missense mutations were identified in NDI patients, most affected highly conserved amino acid residues (positions are encircled in black) (see also Table 3).

The overall identity between full-length mammalian V2R orthologs (36 species) is 85.9±6.7% (see Table 1). During 170 million years of mammalian evolution ∼160 (∼43.1%) out of all amino acid residues in V2R remained unchanged between mammalian species (Figure 2). The overall structural conservation of V2R (84.6±8.0%) is not significantly different when compared with comparable ortholog data sets of other GPCR (20 identical species): rhodopsin (94.3±2.2%), the melanocortin type 4 receptor (MC4R) (93.1±4.8%), the ADP receptor P2Y12 (88.8±5.3% ) and GPR34 (85.4±7.5% ) [32]–[34].

**Table 1:** Structural comparison of mammalian V2R orthologs.

Domain	Length (Min/Max)	Conservation between mammalian orthologs (% Identity)
Full length	365/381	85.9±6.7
N terminus	35/40	67.2±12.3
C terminus	43/48	80.9±9.6
ECL1	15	91.5±5.6
ECL2	24/26	78.8±12.4
ECL3	15	84.0±10.4
ICL1	14/15	84.5±8.7
ICL2	23/24	91.1±7.6
ICL3	39/59	80.7±13.2
TMD1	24	87.9±8.4
TMD2	21	96.2±3.7
TMD3	22	99.0±2.4
TMD4	20	94.9±6.2
TMD5	23	94.2±6.1
TMD6	21	97.0±2.5
TMD7	21	97.2±3.6

The amino acid sequence information of 36 full-length V2R orthologs (see Supporting Material Table S1) was used to determine global and more distinct structural parameters. Segment lengths (minimum/maximum) and the structural conservation between mammalian orthologs (given as % identity determined by Clustal W implemented in MegAlign of Lasergene Ver. 7.1.0) are shown. Data are given as mean±S.D.

**Figure 3:**
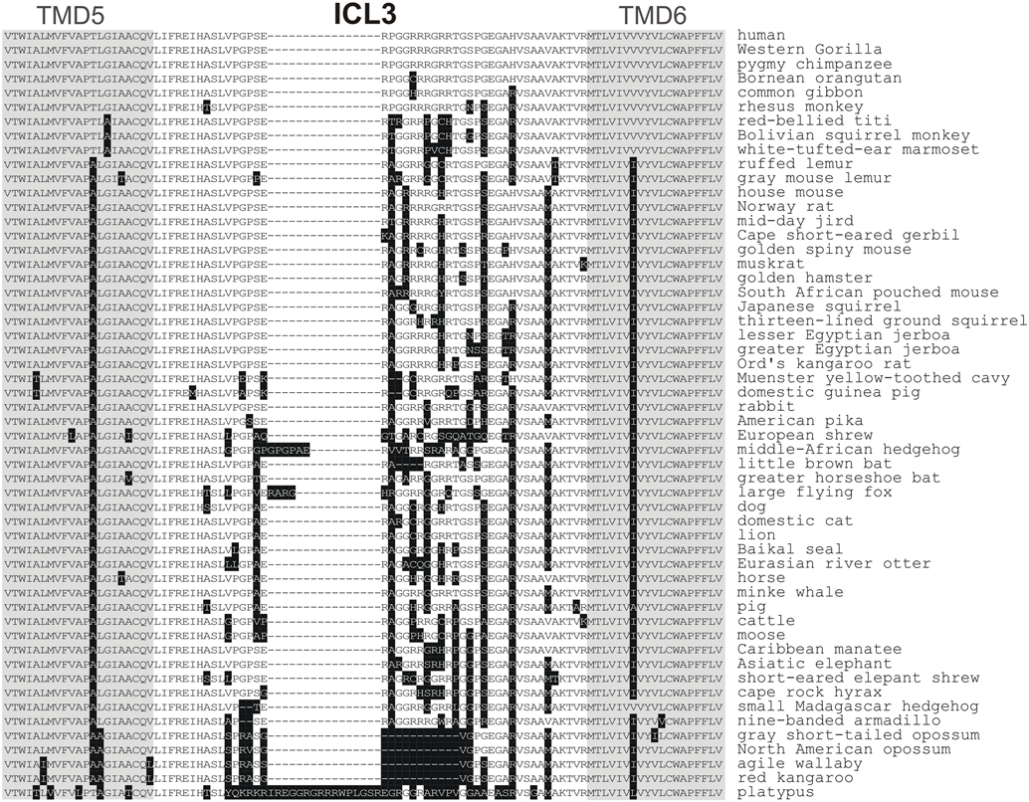
The ICL3 varies between mammalian V2R orthologs. Sequence analysis of V2R orthologs revealed a remarkable length and amino acid variation of ICL3. Positions which differ from the amino acid sequence of human V2R are boxed.

Detailed analysis revealed the following results:

*First*, we found several length variations within the extracellular and intracellular loops (see Table 1). The length variations of the third intracellular loop (ICL3) were most prominent (Figure 3). Although ICL3 has been implicated in G-protein activation, desensitization and interaction with other signalling components in other GPCR, ICL3 of the V2R shows low amino acid sequence conservation. In several species the middle part of ICL3 contains sequence deletions up to 11 amino acids (marsupials) and insertions of 16 amino acids (platypus) when compared to the human V2R. This implicates a rather low functional relevance of the middle portion of the ICL3 in V2R. Indeed, deletion of the respective 11 amino acids in the human V2R (referred to as human del243–253) had no effect on receptor function (Table 2). Additional evidence for a rather low functional relevance of the middle portion of ICL3 comes from split receptors. V2R fragments split in ICL3 reassemble functionally when coexpressed [35]. Further, a naturally occurring 12-bp deletion including amino acid positions 247 to 250 was reported to have no functional relevance in the human V2R [36].

**Table 2:** Functional characterization of mammalian V2R.

Species	basal cAMP (% human V2R)	E_max_ (% human V2R)	EC_50_ (pM)	Cell surface expression# (% human V2R)	Total cellular expression## (% human V2R)
***Primates***					
human	100 (21)	100 (21)	238.3±60.7 (18)	100 (21)	100 (15)
human del243–253	122.4±13.9 (9)	96.3±14.2 (9)	184.8±34.8 (8)	104.9±2.9 (11)	109.1±5.4 (4)
common gibbon	88.6±8.3 (7)	114.2±7.7 (7)	497.3±101.7 (7)	96.7±3.2 (6)	103.2±1.7 (5)
crab-eating macaque	119.7±7.0 (8)	120.5±16.9 (8)	760.0±187.6 (8)	102.1±6.4 (9)	93.8±3.0 (5)
African green monkey	94.9±18.6 (7)	78.3±13.5 (7)	266.7±53.9 (7)	98.1±5.7 (10)	106.6±5.2 (4)
***Rodentia***					
house mouse	106.6±12.0 (14)	102.7±8.3 (14)	231.8±47.6 (14)	89.1±4.5 (11)	108.5±3.2 (9)
Algerian grass mouse	114.0±12.2 (8)	91.4±14.0 (8)	368.6±76.5 (8)	90.9±3.3 (8)	111.6±3.6 (5)
muskrat	111.3±8.6 (9)	87.4±8.3 (9)	211.7±65.9 (8)	74.3±2.6 (7)	107.9±1.8 (4)
golden hamster	101.7±24.8 (6)	94.2±17.4 (6)	175.3±36.2 (6)	92.8±4.5 (8)	108.6±3.5 (7)
Northern mole vole	78.7±13.7 (10)	69.9±11.1 (10)	386.4±91.0 (10)	96.6±4.2 (6)	103.7±3.9 (5)
steppe lemming	107.9±24.7 (8)	86.2±18.9 (8)	324.2±92.1 (8)	100.5±4.6 (8)	108.1±3.1 (7)
bushy-tailed jird	118.8±26.6 (7)	46.1±7.4 (7)*	404.4±65.2 (7)	58.7±6.8 (10)	103.0±3.1 (5)
Mongolian gerbil	108.9±10.0 (8)	93.8±11.4 (8)	268.1±53.4 (7)	91.1±3.4 (7)	106.8±5.5 (3)
Wagner's gerbil	124.2±24.0 (6)	63.5±11.9 (7)*	265.9±80.7 (7)	71.5±4.5 (7)	104.6±4.4 (5)
large naked-sole gerbil	106.5±16.9 (6)	108.3±10.1 (6)	216.3±39.9 (6)	90.3±3.3 (8)	101.2±5.6 (4)
lesser Egyptian jerboa	127.7±17.2 (8)	110.3±12.9 (8)	678.7±99.7 (8)	101.1±4.7 (7)	102.9±2.2 (7)
greater Egyptian jerboa	109.9±20.3 (7)	98.2±12.3 (7)	738.7±172.6 (7)	102.5±6.3 (8)	110.1±2.4 (5)
Japanese squirrel	97.2±11.0 (10)	91.8±6.7 (10)	470.4±84.2 (10)	83.2±3.4 (5)	96.1±4.4 (4)
***Carnivora***					
lion	92.1±7.2 (20)	95.7±11.4 (20)	586.0±74.5 (20)	73.9±6.3 (10)	93.1±5.3 (9)
dog	101.8±7.1 (10)	124.6±13.8 (10)	841.3±232.7 (10)	95.0±3.2 (7)	88.8±7.3 (5)
Eurasian river otter	107.0±15.0 (15)	91.9±12.7 (15)	306.9±64.2 (15)	83.4±4.8 (8)	103.8±1.6 (9)
Baikal seal	113.0±8.0 (14)	74.9±6.3 (14)	131.5±40.6 (14)	67.4±5.2 (7)	103.8±1.7 (9)
Californian sea lion	105.6±8.1 (14)	70.0±6.2 (14)	114.8±41.3 (14)	65.1±8.9 (7)	101.8±2.5 (9)
***Cetacea***					
minke whale	111.6±22.6 (7)	62.1±10.1 (7)*	294.9±77.8 (6)	37.0±5.8 (8)	86.1±12.7 (7)
***Proboscidea***					
Asiatic elephant	117.7±18.0 (8)	79.7±19.4 (8)	210.6±65.1 (8)	55.1±5.6 (6)	99.3±3.1 (5)
African savanna elephant	109.6±18.2 (9)	78.3±15.1 (9)	300.8±86.3 (9)	49.3±4.5 (7)	96.7±2.0 (8)
***Sirenia***					
Caribbean manatee	115.7±18.7 (13)	53.7±6.8 (13)*	147.2±25.5 (13)	31.7±3.4 (7)	101.5±3.0 (5)
***Macroscelidea***					
short-eared elephant shrew	102.3±26.0 (8)	81.1±18.6 (8)	375.7±53.6 (8)	67.7±3.5 (6)	81.3±12.4 (6)
***Artiodactyla***					
cattle	101.0±6.5 (16)	62.1±7.1 (16)*	154.0±43.0 (15)	56.5±7.2 (11)	78.6±6.0 (9)
***Marsupials***					
***Didelphimorphia***					
North American opossum	224.9±66.7 (8)	168.1±35.4 (8)	483.8±207.9 (5)	106.5±9.9 (7)	99.1±1.0 (6)
***Diprotodontia***					
red kangaroo	433.0±65.6 (8)*	193.5±27.9 (8)*	340.0±107.6 (5)	129.0±6.8 (7)	98.5±1.5 (6)
agile wallaby	340.6±49.4 (8)*	185.1±20.6 (9)*	340.1±81.6 (6)	123.0±5.7 (8)	97.5±2.2 (7)
long-nosed potoroo	314.2±65.8 (9)*	107.3±22.0 (9)	256.1±118.2 (6)	57.3±6.0 (8)	96.3±1.7 (7)

For functional characterization COS-7 cells were transiently transfected with V2R constructs and non-radioactive cAMP assays were performed as described in *Materials and Methods*. E_max_ and EC_50_ values were determined from concentration-response-curves of AVP (1 fM–10 µM) using GraphPad Prism. Data are presented as mean±S.E.M. of independent experiments (number indicated in parentheses), each carried out in duplicate. Cyclic AMP levels of non-stimulated (10.0±2.2 amol/cell) and 10 µM AVP-stimulated (185.9±28.6 amol/cell) human V2R served as reference basal and E_max_ values and were set 0% and 100%, respectively. Cell surface and total expression levels of V2R orthologs were measured by cell surface and total cellular ELISAs (see *Materials and Methods*). Specific optical density (OD) readings (OD value of HA-tagged construct minus OD value of GFP-transfected cells) are given as a percentage of human double HA/FLAG-tagged V2R.

# For the cell surface ELISA the non-specific OD value (GFP) was 0.013±0.007 (set 0%) and the OD value of the human V2R was 0.983±0.133 (set 100%).

## For the total cellular ELISA the non-specific OD value (GFP) was 0.061±0.020 (set 0%) and the OD value of the human V2R was 0.814±0.118 (set 100%). The number of independent experiments, each carried out in quadruplicate, is given in parentheses.

* indicates significance differences (p<0.01) to the average basal and AVP-induced activity of all V2R orthologs.

*Second*, the N terminus is most diverse in its length and amino acid sequence between mammalian orthologs (Table 1) clearly indicating less specific relevance in ligand binding and in receptor activity.

*Third*, the C terminus of the human V2R contains two Cys residue (Cys^341^, Cys^342^) which allows anchoring via palmitoylation and forming a fourth intracellular loop (ICL4). It was demonstrated by mutagenesis of the two Cys residues that palmitoylation of V2R is important for intracellular trafficking and/or sequestration/internalization. Most efficient reduction of receptor cell surface expression was found when Cys^341^ alone or both Cys residues were mutated [37]. Consistently, Cys at position 341 is 100% conserved in mammals whereas Cys^342^ is substituted by Arg, Trp and Phe in several mammalian species.

*Fourth*, a proposed glutamate/dileucine motif equivalent (E^335^LRSLL^340^ in human V2R) in the C terminus [38] is highly preserved during mammalian V2R evolution. The motif may represent a transport signal from the endoplasmic reticulum to the Golgi apparatus. Leu^339^ was found substituted by Trp and Leu^340^ by Phe in some V2R orthologs keeping with the hydrophobic nature of ICL4 [39].

*Fifth*, most rhodopsin-like GPCR possess an evolutionarily conserved Asp-Arg-Tyr (DRY) motif in the C-terminal region of TMD3. Mutations of the first two residues within this motif usually alter receptor function and, when they occur naturally, can even cause diseases. In V2R the DRY motif is a DRH which is highly conserved in mammals and only the manatee has DRQ. Variation of the Tyr residue in the DRY motif appears to be functionally tolerated since this position is variable in many GPCR and substitution of Tyr has no or marginal effects in most cases [40].

*Sixth*, phosphorylation plays a pivotal role in the regulation of GPCR function. Previous studies identified putative sites which are clustered in the C terminus and in ICL3 and are phosphorylated by specific GPCR kinases (GRK) and by protein kinase A (PKA), respectively [41]–[43]. Whereas abundant putative Ser/Thr phosphorylation sites are found in the C terminus of all V2R orthologs the putative PKA phosphorylation site within ICL3 at Ser^255^ [41] is substituted by Asn, Gly and Asp in many V2R orthologs implicating that this position is, if at all, a substrate for PKA in only a few species.

### Evaluation of naturally occurring mutations in the human V2R

As stated above, several NDI patients carrying an inactivating V2R mutation managed the disease by sufficient fresh water supply without any medications. One can speculate that V2R function is less important when a species lives in a water-*ad-libitum* environment. Therefore, we analyzed whether NDI-causing (inactivating) variants of the human V2R naturally occur in other mammalian V2R orthologs. To date, about 113 missense variants of the human V2R at 90 amino acid positions have been described (see Supporting Material Table S3) [31], [44]. Out of these missense variants 91% are assumed to be NDI causing. We correlated this information with the evolutionary variability at the respective amino acid position (Figure 2, Table 3). About 91% of all NDI-causing mutations hit positions that are fully conserved or vary between two amino acids (Table 3). None of the NDI-causing variants was found in any of the mammalian V2R orthologs. Therefore, our analysis provided no evidence of inactivating mutations in the mammalian V2R orthologs investigated.

**Table 3:** Function-conservation relationship of natural occurring missense mutations in V2R.

Variability of the position	Wild-type function	NDI causing
>2 amino acids	4	9
2 amino acids	4	11
1 amino acid	2	83

More than 113 missense mutations at 90 positions within the human V2R have been described (see Figure 2). About 91% of all V2R missense variants identified so far are NDI causing. The table gives a correlation between the functional consequence of individual mutations and the evolutionary conservation of the mutated position. The complete list of missense mutations included in the analysis and the references are given in Supporting Material Table S3. The evolutionary conservation of each mutated position was determined by aligning V2R orthologs available and the variability of the position was categorized in three groups from no variation (1 amino acid) to high variation (>2 amino acids) in mammalian orthologs.

### Adaptive gain-of-function in marsupial V2R orthologs

Conservation and variability of distinct positions can only be properly interpreted in the light of functional data of different orthologs. Therefore, a number of mammalian V2R orthologs were cloned, expressed in COS-7 cells, stimulated with arginine vasopressin (AVP) and functionally analyzed in cAMP assays. As shown in Table 2, AVP potencies (EC_50_) at the selected V2R orthologs varied between 115 pM (Californian sea lion) and 841 pM (dog) and were in a comparable range to findings with the human V2R (238 pM). There is no obvious correlation of the agonist potencies to the environmental conditions of the respective species. Our data may reflect the naturally tolerated range of vasopressin potency between roughly 0.1 and 1 nM. Consistently, there is no report where an exclusive increase in EC_50_ values of lower than one order of magnitude causes NDI.

Species differences were found for the efficacy of receptor signaling. Several aquatic mammals (Baikal seal, Californian sea lion, minke whale, Caribbean manatee), cattle and some gerbils (Wagner's gerbil, bushy-tailed jird) displayed reduced E_max_ values when compared with the human V2R (see Table 2). The reduced E_max_ values are most likely caused by reduction in cell surface expression levels of these V2R orthologs. For the Baikal seal V2R, one can discuss this as some reduction of constraint because of free access to fresh water and, therefore, as a reduced necessity to concentrate the urine. However, the reduced efficacy of V2R signal transduction in marine mammals (Californian sea lion, minke whale, Caribbean manatee) and gerbils is unexpected because these animals produce highly concentrated urine, which is especially important for mammals in a hyperosmotic and desert environment, respectively. Variations in kidney morphology observed in marine mammals does not appear to afford them any greater benefit than terrestrial mammals, suggesting that the adaptation of mammals to sea water was accomplished also via hormonal regulation of urine concentration and/or the rate of urine formation [45]. The anti-diuretic function of AVP in marine mammals is still inconclusive. AVP levels in dolphins and seals are rather low despite high urine osmolality [45]. In sum, it is unlikely that the reduced signaling efficacy of V2R in some aquatic mammals and gerbils is a result of evolutionary adaptation. It rather reflects the naturally occurring spectrum of V2R function or a reduced constraint at the V2R because of other adaptive mechanisms to concentrate the urine.

**Figure 4:**
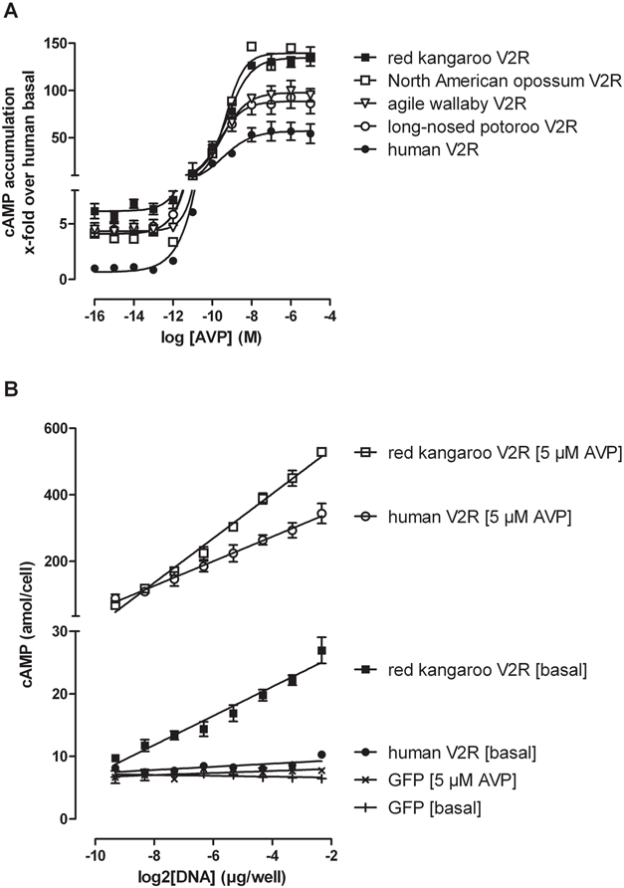
Increased basal activity of marsupial V2R orthologs. For functional characterization of the human and marsupial V2R orthologs, the respective expression plasmids were transfected into COS-7 cells and tested for AVP-induced cAMP accumulation. A) 48 hours after transfection cells were stimulated with increasing concentrations of AVP. Intracellular cAMP was measured with AlphaScreen cAMP assay (see *Materials and Methods*). B) To further assess the increased basal activity of marsupial V2R orthologs increasing amounts of plasmid DNA from the red kangaroo ortholog were transfected and cAMP assays were performed. As expected for a constitutive active receptor, basal receptor activity correlates with the amount of transfected plasmid DNA. Data are given as mean±S.E.M. of three experiments each performed in duplicate.

Striking differences were found in the basal activity of marsupial V2R orthologs. All marsupial V2R showed elevated basal activity ranging from 2.2 to 4.3-fold over the human and all other V2R orthologs tested (Figure 4A, Table 2). The orthologs of arid-adapted species, red kangaroo and agile wallaby, displayed the highest basal activity and a significant gain in receptor efficiency. ELISA studies exclude that the increased basal activity was due to higher cell surface expression levels. Basal cAMP levels displayed strong correlation to the transfected plasmid DNA indicating genuine constitutively active receptors (Figure 4B). The increase in basal activity of e.g. the red kangaroo V2R was comparable to basal cAMP levels found in patients with NSIAD [19]. Besides basal activity, marsupial V2R displayed normal V2R function and cellular expression levels (Table 2). When compared to all other mammalian V2R orthologs tested the kangaroo V2R contains 23 positions with amino acids that are unique to marsupial orthologs (see Supporting Material Figure S2). Thirteen of them are at positions which are 100% conserved among all other non-marsupial V2R orthologs. Unfortunately, there is no obvious individual position known to cause constitutive activity when substituted. It is more likely, that sequential or combinatorial substitutions at the sequence background of the marsupial V2R orthologs lead to increased basal receptor function. This hypothesis is supported by the fact that constitutive activity differed significantly between marsupial V2R orthologs (see Table 2).

Several marsupials, like the red kangaroo and the agile wallaby, are adapted to extremely dry climates. Most marsupials display a typical mammalian pattern of hormonal control of kidney function and water excretion, with plasma vasopressin levels correlating highly with the urine/plasma osmolality ratio [46]. However, their renal ability to reabsorb water and concentrate the urine is extraordinary high. For example, hare wallaby and red kangaroo have average urine osmolalities between 1,843 and 2,357 mosmol kg^−1^ [47] and red kangaroo can concentrate up to 4,000 mosmol kg^−1^ [48]. For comparison, average human urine osmolality is 800 mosmol kg^−1^ and human kidneys can concentrate the urine up to 1,400 mosmol kg^−1^. Although some studies suggest that mammals with relatively long loops of Henle for their body size tend to have greater than average urinary concentrating ability [49], [50], detailed analyses found no relationship between urine osmolality and absolute length of the loop of Henle [4], [51]. Moreover, a recent report found that one desert wallaby (*Petrogale rothschildi*) appears to be unique amongst mammals lacking antidiuretic response to vasopressin [47]. Therefore, other factors than morphological and hormonal must contribute to adaption of the renal water reabsorption to the different water accessibilities. An increased agonist-independent V2R function, as found for marsupials, can be interpreted as a shift in the set point of the renal-neurohypophysial hormone circuit to realize sufficient water reabsorption already at low hormone levels. Similarly, increased basal activity is physiologically found also for the thyroid stimulating hormone (TSH) receptor, the MC1R and the MC4R to assure basal thyroid hormone production, pigmentation and energy homeostasis, respectively [52]–[54]. One may argue that increased basal V2R activity in marsupials should have pathophysiological consequences as found in NSIAD patients. However, serum hypoosmolality in these patients results from normal alimentary water intake but inadequate renal water elimination. In contrast to humans one can not expect an *ad libitum* supply of water for desert marsupials. Therefore, serum hypoosmolality, as observed in NSIAD patients carrying an activating V2R mutation, is probably unlikely. This example nicely demonstrates the biological interpretation of a specific protein function (e.g. basal activity) as an advantage (adaptation) or a disadvantage (disease) depending on environmental conditions.

### Conclusion

The V2R is a key component in regulating the renal water reabsorption but its contribution to adaptation of mammals to different water accessibility was not investigated yet. We found that the mammalian V2R is highly conserved in its amino acid structure and functional properties. Although some mammals have unlimited access to fresh water there is no evidence for complete loss of V2R function in those or any other mammals investigated. This indicates that some V2R activity is essentially required for species survival. In contrast, structural changes in the marsupial V2R resulted in a gain of V2R function and may provide an advantage to maintain water and electrolyte homeostasis for those marsupials living in arid habitats.

## Materials and Methods

### V2 vasopressin receptor ortholog identification and site-directed mutagenesis

To analyze the sequence of V2R orthologs, genomic DNA samples were prepared from tissue or peripheral mononuclear blood cells of various mammalian species (sources are given in Supporting Material Table S1). Tissue samples were digested in lysis buffer (50 mM Tris/HCl, pH 7.5, 100 mM EDTA, 100 mM NaCl, 1% SDS, 0.5 mg/ml proteinase K) and incubated at 55°C for 18 hours. DNA was purified by phenol/chloroform extraction and ethanol precipitation. Degenerated primer pairs (Supporting Material Table S2) were applied to amplify V2R specific sequences. PCR reactions were performed with *Taq* polymerase under variable annealing and elongation conditions. Specific PCR products were directly sequenced and/or subcloned for sequencing into the pCR2.1-TOPO vector (Invitrogen, La Jolla, CA). Sequencing reactions were performed with a dye-terminator cycle sequencing kit (Applied Biosystems) on an ABI 3700 automated sequencer (Applied Biosystems). Based on considerable sequence similarities of the 5′- and 3′-untranslated regions (UTR) of V2R genes primers were designed (Supporting Material Table S2) which allowed for the identification of some sequences encoding the N and C termini of mammalian V2R orthologs.

Full-length V2R were inserted into the mammalian expression vector pcDps (Schöneberg et al 1996) and epitope-tagged with an N-terminal hemagglutinin (HA) epitope and a C-terminal FLAG-tag by a PCR-based overlapping fragment mutagenesis approach. For most species only genomic DNA was available for V2R ortholog cloning. Therefore, most mammalian V2R constructs were build by inserting a genomic DNA fragment containing the second intron (encompassing amino acid position 68 (2.33, numbering of the relative position within GPCR [55]) to amino acid position 321 (7.49) of the respective mammalian ortholog into the double-tagged human V2R-pcDps expression plasmid keeping the human N and C termini. Identity of all constructs and correctness of all PCR-derived sequences were confirmed by restriction analysis and sequencing. To assure that these constructs are functionally equivalent to expression constructs of the full-length cDNA, the different constructs were compared in cAMP assays. As shown in Supporting Material Figure S1 function of cDNA constructs (dog, bovine) were identical to constructs containing the TMD core and second intron of the respective species flanked by the human N and C termini.

### Cell culture and functional assays

COS-7 cells were grown in DMEM supplemented with 10% fetal bovine serum, 100 U/ml penicillin, and 100 µg/ml streptomycin at 37°C in a humidified 7% CO_2_ incubator. Lipofectamine™ (Invitrogen) was used for transient transfection of COS-7 cells. Thus, cells were split into 50-ml cell culture flasks (1×10^6^ cells per flask) and transfected with 4 µg of plasmid DNA/flask.

#### ALPHAScreen™ cAMP assay

cAMP content of cell extracts was determined by a non-radioactive cAMP accumulation assay based on the ALPHAScreen™ technology according to the manufacturer's protocol (Perkin Elmer LAS, Rodgau-Jügesheim, Germany) [56]. One day after transfection cells were split into 48-well plates (5×10^4^ cells/well). Stimulation with various agonist concentrations ([Arg8]-vasopressin acetate salt from Sigma-Aldrich, Seelze, Germany) was performed 48 h after transfection for 1 h at 37°C. Reactions were stopped by aspiration of media and cells were lysed in 50 µl of lysis buffer containing 1 mM 3-isobutyl-1-methylxanthine. From each well 5 µl of lysate were transferred to a 384-well plate. Acceptor beads (in stimulation buffer without 3-isobutyl-1-methylxanthine) and donor beads were added according to manufacturers' protocol. Cyclic AMP accumulation data were analyzed using GraphPad Prism version 5.01 for Windows (GraphPad Software, San Diego, California, USA).

#### ELISA studies

To estimate cell surface expression of receptors carrying an N-terminal HA-tag, we used an indirect cellular ELISA [57]. To further assess the amounts of full-length double-tagged V2Rs, a sandwich ELISA was used essentially as described previously [17]. Briefly, microtiter plates were coated with a polyclonal anti-FLAG antibody and cell lysates were applied. Following intensive washing with PBS containing 0.05% Triton X-100 (PBS-T), plates were incubated with a peroxidase-labeled monoclonal anti-HA antibody (12CA5; 1 mg/mL PBS-T). After removal of excess unbound conjugate, H_2_O_2_ and o-phenylenediamine (2.5 mmol/L each in 0.1 mol/L phosphate/citrate buffer, pH 5.0) were added to serve as substrate and chromogen, respectively. After 15 min, the enzyme reaction (carried out at room temperature) was stopped by the addition of 1 mol/L H_2_SO_4_ containing 0.05 mol/L Na_2_SO_3_, and colour development was measured bichromatically at 492 and 620 nm using an ELISA reader (Sunrise™, Tecan Group Ltd.).

### Data deposition

The sequences reported in this paper have been deposited in the GeneBank database (accession no. FJ411185-FJ411251; Supporting Material Table S1).

## Supporting Information

Figure S1 Equivalence of cDNA and TMD core constructs.
(0.12 MB PDF)

Figure S2 Structural differences between marsupial and non-marsupial V2R orthologs.
(0.43 MB PDF)

Table S1 Description, database accession and sources of genomic DNA samples, where applicable.
(0.04 MB PDF)

Table S2 Primers used for V2 ortholog amplification and cloning.
(0.01 MB PDF)

Table S3 Missense mutations found in human V2R.
(0.01 MB PDF)
