# Supplementary material for: Involvement of the V2 Vasopressin Receptor in Adaptation to Limited Water Supply
Source: PLoS One. 2009 May 18;4(5):e5573. doi: 10.1371/journal.pone.0005573 (PMC2680020; doi:10.1371/journal.pone.0005573)
Supplement: Figure S1 — Equivalence of cDNA and TMD core constructs. (0.12 MB PDF) [file pone.0005573.s001.pdf]

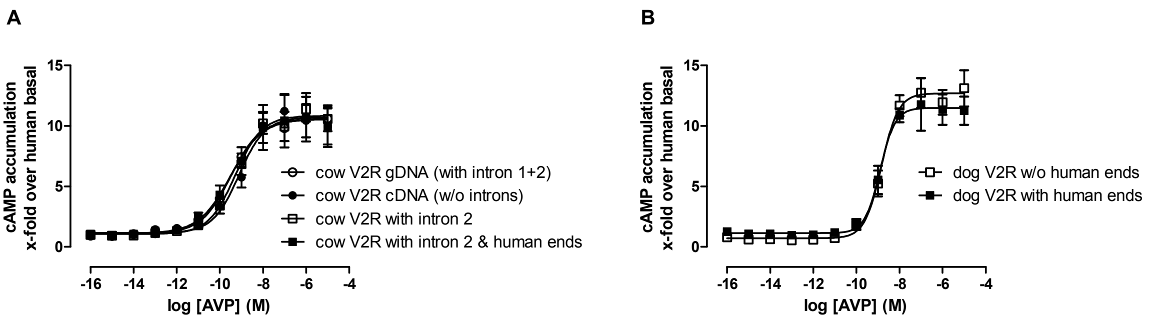

Figure S1. Equivalence of cDNA and TMD core constructs.
We tested whether the presence of introns and the N and C termini of the human V2R have an impact on ortholog function when compared to constructs containing the full-length cDNA. As shown for bovine (A) and dog (B) orthologs these modifications have no effect on basal and stimulated cAMP production.
