# Supplementary material for: Involvement of the V2 Vasopressin Receptor in Adaptation to Limited Water Supply
Source: PLoS One. 2009 May 18;4(5):e5573. doi: 10.1371/journal.pone.0005573 (PMC2680020; doi:10.1371/journal.pone.0005573)
Supplement: Figure S2 — Structural differences between marsupial and non-marsupial V2R orthologs. (0.43 MB PDF) [file pone.0005573.s002.pdf]

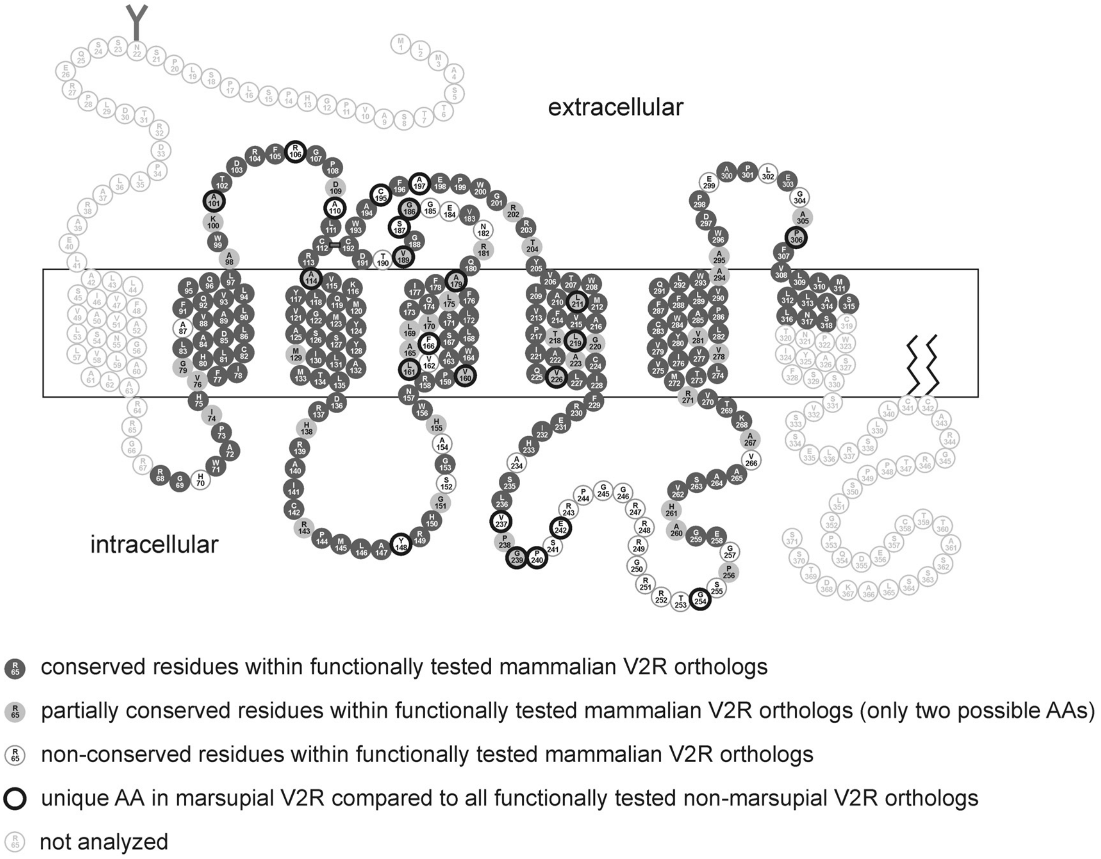

Figure S2. Structural differences between marsupial and non-marsupial V2R orthologs. To identify residues responsible for increased basal activity in kangaroo V2R amino acid sequences of all functionally tested V2R orthologs (see Table 3) were compared. Amino acid residues at 23 positions were unique to marsupial orthologs.
