# Supplementary material for: Involvement of the V2 Vasopressin Receptor in Adaptation to Limited Water Supply
Source: PLoS One. 2009 May 18;4(5):e5573. doi: 10.1371/journal.pone.0005573 (PMC2680020; doi:10.1371/journal.pone.0005573)
Supplement: Table S1 — Description, database accession and sources of genomic DNA samples, where applicable. (0.04 MB PDF) [file pone.0005573.s003.pdf]

Table S1. Description, database accession and sources of genomic DNA samples, where applicable.

common name	species	cloned sequence	NCBI accession /source	source of genomic DNA
**Eutheria** ***Primates***				
human	*Homo sapiens*	Ex1-Ex2-Ex3	NCBI accession: FJ411207; AY242131.1, NG_008687	own source
Western gorilla	*Gorilla gorilla*	pEx2-Ex3	NCBI accession: FJ411206	Dr. W. Enard, MPI, Leipzig, Germany
chimpanzee	*Pan troglodytes*	Ex1-Ex2-Ex3	NCBI *Pan troglodytes* trace archive: gnl|ti|1679876518 and others	-
pygmy chimpanzee, bonobo	*Pan paniscus*	pEx2-pEx3	NCBI accession: FJ411236	Dr. W. Enard, MPI, Leipzig, Germany
Bornean orangutan	*Pongo pygmaeus*	Ex1-Ex2-Ex3	NCBI accession: FJ411239; NCBI *Pongo pygmaeus abelii* trace archive: gnl|ti|873465220 and others	Dr. W. Enard, MPI, Leipzig, Germany
common gibbon	*Hylobates lar*	pEx2-pEx3	NCBI accession: FJ411208, FJ411209	Dr. W. Enard, MPI, Leipzig, Germany
white-cheeked gibbon	*Nomascus leucogenys*	Ex1-Ex2-Ex3	NCBI *Nomascus leucogenys* trace archive: gnl|ti|2055162064 and others	-
silvered leaf monkey	*Presbytis cristata*	pEx2-pEx3	NCBI accession: FJ411241	Dr. W. Enard, MPI, Leipzig, Germany
guereza	*Colobus guereza*	Ex1-Ex2-Ex3	NCBI accession: FJ411196; NCBI *Colobus guereza* trace archive: gnl|ti|1645319750 and others	Dr. W. Enard, MPI, Leipzig, Germany
African green monkey	*Chlorocebus aethiops (Cercopithecus aethiops)*	Ex1-Ex2-Ex3	NCBI accession: FJ411195; NCBI *Cercopithecus aethiops* trace archive: gnl|ti|1941073555 and others	ATCC - cell collection, COS-7
hamadryas baboon	*Papio hamadryas*	Ex1-Ex2-Ex3	NCBI accession: FJ411237; NCBI *Papio hamadryas* trace archive: gnl|ti|1924193977 and others	Dr. W. Enard, MPI, Leipzig, Germany
olive baboon	*Papio anubis*	Ex1-Ex2-Ex3	NCBI *Papio anubis* trace archive: gnl|ti|441860415 and others	-
rhesus monkey	*Macaca mulatta*	Ex1-Ex2-Ex3	NCBI *Macaca mulatta* trace archive: gnl|ti|1002597000 and others; NCBI accession: XM_001088539.1	-
crab-eating macaque	*Macaca fascicularis*	pEx2-pEx3	NCBI accession: FJ411218	Dr. W. Enard, MPI, Leipzig, Germany
mandrill	*Mandrillus sphinx*	pEx2-pEx3	NCBI accession: FJ411220	Dr. W. Enard, MPI, Leipzig, Germany
Bolivian squirrel monkey	*Saimiri boliviensis*	Ex1-Ex2-Ex3	NCBI *Saimiri boliviensis boliviensis* trace archive: gnl|ti|1906784044 and others	-

common name	species	cloned sequence	NCBI accession /source	source of genomic DNA
white-tufted-ear marmoset	*Callithrix jacchus*	Ex1-Ex2-Ex3	NCBI *Callithrix jacchus* trace archive: gnl|ti|509569134 and others	-
red-bellied titi	*Callicebus moloch*	Ex1-Ex2-Ex3	NCBI *Callicebus moloch* trace archive: gnl|ti|1458887602 and others	-
Ma's night monkey	*Aotus nancymaae*	Ex1-Ex2-Ex3	NCBI *Aotus nancymaae* trace archive: gnl|ti|1493846453 and others	-
gray mouse lemur	*Microcebus murinus*	Ex1-Ex2-Ex3	NCBI *Microcebus murinus* trace archive: gnl|ti|1286200793 and others	-
ruffed lemur	*Varecia variegata*	pEx2-pEx3	NCBI accession: FJ411249	Dr. W. Enard, MPI, Leipzig, Germany
ring-tailed lemur	*Lemur catta*	pEx2-pEx3	NCBI accession: FJ411214	Dr. W. Enard, MPI, Leipzig, Germany
small-eared galago	*Otolemur garnettii*	pEx2	NCBI *Otolemur garnettii* trace archive: gnl|ti|2046409070 and others	-
Scandentia				
northern tree shrew	*Tupaia belangeri*	Ex1-Ex2-Ex3	NCBI *Tupaia belangeri* trace archive: gnl|ti|1047006132 and others	-
Rodentia				
house mouse	*Mus musculus*	Ex1-Ex2-Ex3	NCBI accession: FJ411230, FJ411231, NM_019404.1, AJ006691 ; NCBI *Mus musculus* trace archive: gnl|ti|932475338 and others;	Dr. A. Orth, University of Montpellier, France
Norway rat	*Rattus norvegicus*	Ex1-Ex2-Ex3	NCBI *Rattus norvegicus* trace archive: gnl|ti|69654380 and others; NCBI accession: NM_019136.1	-
Algerian striped grass mouse	*Lemniscomys barbarus barbarus*	pEx2-pEx3	NCBI accession: FJ411213	Dipl.-Biol. Christian Kern, Tierpark Berlin, Berlin, Germany
Namaqua rock mouse	*Aethomys namaquensis*	pEx2-pEx3	NCBI accession: FJ411186	Dipl.-Biol. Christian Kern, Tierpark Berlin, Berlin, Germany
mid-day jird	*Meriones meridianus*	pEx2-pEx3	NCBI accession: FJ411223	Dr. C. Pitra, IZW, Berlin, Germany
Kara-Kum mid-day jird	*Meriones meridianus penicilliger*	pEx2-pEx3	NCBI accession: FJ411224	Dipl.-Biol. Christian Kern, Tierpark Berlin, Berlin, Germany
lesser Shaw's Jird	*Meriones shawi isis*	pEx2-pEx3	NCBI accession: FJ411225, FJ411226, FJ411227	Dipl.-Biol. Christian Kern, Tierpark Berlin, Berlin, Germany
Mongolian gerbil	*Meriones unguiculatus*	pEx2-pEx3	NCBI accession: FJ411228	Research Facility for Experimental Medicine, Berlin, Germany

**common name**	**species**	**cloned sequence**	**NCBI accession /source**	**source of genomic DNA**
Wagner's gerbil	*Gerbillus dasyurus*	pEx2-pEx3	NCBI accession: FJ411203	Dipl.-Biol. Christian Kern, Tierpark Berlin, Berlin, Germany
lesser Egyptian gerbil	*Gerbillus gerbillus*	pEx2-pEx3	NCBI accession: FJ411204, FJ411205	Dipl.-Biol. Christian Kern, Tierpark Berlin, Berlin, Germany
bushy-tailed jird	*Sekeetamys calurus*	pEx2-pEx3	NCBI accession: FJ411244	Dipl.-Biol. Christian Kern, Tierpark Berlin, Berlin, Germany
fat-tailed gerbil	*Pachyuromys duprasi*	pEx2-pEx3	NCBI accession: FJ411234	Dipl.-Biol. Christian Kern, Tierpark Berlin, Berlin, Germany
Cape short-eared gerbil	*Desmodillus auricularis*	pEx2-pEx3	NCBI accession: FJ411197	Dipl.-Biol. Christian Kern, Tierpark Berlin, Berlin, Germany
large naked-sole gerbil	*Tatera sp.*	pEx2-pEx3	NCBI accession: FJ411246	Dipl.-Biol. Christian Kern, Tierpark Berlin, Berlin, Germany
golden spiny mouse	*Acomys russatus russatus*	pEx2-pEx3	NCBI accession: FJ411185	Dipl.-Biol. Christian Kern, Tierpark Berlin, Berlin, Germany
pouched mouse	*Saccostomus campestris*	pEx2-pEx3	NCBI accession: FJ411242	Dipl.-Biol. Christian Kern, Tierpark Berlin, Berlin, Germany
golden hamster	*Mesocricetus auratus*	pEx2-pEx3	NCBI accession: FJ411229	Dipl.-Biol. Christian Kern, Tierpark Berlin, Berlin, Germany
Mongolian silver vole	*Alticola semicanus alleni*	pEx2	NCBI accession: FJ411189, FJ411190	Dipl.-Biol. Christian Kern, Tierpark Berlin, Berlin, Germany
Northern mole vole	*Ellobius talpinus*	pEx2-pEx3	NCBI accession: FJ411201	Dipl.-Biol. Christian Kern, Tierpark Berlin, Berlin, Germany
steppe lemming	*Lagurus lagurus*	pEx2-pEx3	NCBI accession: FJ411212	Dipl.-Biol. Christian Kern, Tierpark Berlin, Berlin, Germany
muskrat	*Ondatra zibethicus*	pEx2-pEx3	NCBI accession: FJ411232, FJ411233	Dr. Lauenstein, Federal Centre for Agriculture and Forestry, Braunschweig, Germany
greater Egyptian jerboa	*Jaculus orientalis*	pEx2-pEx3	NCBI accession: FJ411211	Dipl.-Biol. Christian Kern, Tierpark Berlin, Berlin, Germany
lesser Egyptian jerboa	*Jaculus jaculus vocator*	pEx2-pEx3	NCBI accession: FJ411210	Dipl.-Biol. Christian Kern, Tierpark Berlin, Berlin, Germany
thirteen-lined ground squirrel	*Spermophilus tridecemlineatus*	Ex1-Ex2-Ex3	NCBI *Spermophilus tridecemlineatus* trace archive: gnl|ti|1038568813 and others	-

common name	species	cloned sequence	NCBI accession /source	source of genomic DNA
Japanese squirrel	*Sciurus lis*	pEx2-pEx3	NCBI accession: FJ411243	Dipl.-Biol. Christian Kern, Tierpark Berlin, Berlin, Germany
Ord's kangaroo rat	*Dipodymus ordii*	Ex1-Ex2-Ex3	NCBI *Dipodymus ordii* trace archive: gnl|ti|1567934981 and others	-
Spinifex hopping mouse	*Notomys alexis*	pEx2, pEx3	NCBI accession: AY856059	-
domestic guinea pig	*Cavia porcellus*	Ex1-Ex2-Ex3	NCBI *Cavia porcellus* trace archive: gnl|ti|1606482338 and others	-
Muenster yellow-toothed cavy	*Galea monasteriensis*	pEx2-pEx3	NCBI accession: FJ411202	Dipl.-Biol. Christian Kern, Tierpark Berlin, Berlin, Germany
Lagomorpha				
American pika	*Ochotona princeps*	pEx2-Ex3	NCBI *Ochotona princeps* trace archive: gnl|ti|1547255439 and others	-
rabbit	*Oryctolagus cuniculus*	Ex1-Ex2-Ex3	NCBI *Oryctolagus cuniculus* trace archive: gnl|ti|2052700682 and others	-
Cetartiodactyla				
minke whale	*Balaenoptera acutorostrata*	pEx2-pEx3	NCBI accession: FJ411191, FJ411192	Dr. P. J. Palsboll, University of Wales, U.K.
bottlenosed dolphin	*Tursiops truncatus*	Ex1-Ex2-Ex3	NCBI *Tursiops truncatus* trace archive: gnl|ti|1474207802 and others	-
pig	*Sus scrofa*	Ex1-Ex2-Ex3	NCBI accession: FJ411245; NM_214232; NCBI *Sus scrofa* trace archive: gnl|ti|2107641237 and others	own source
cattle	*Bos taurus*	Ex1-Ex2-Ex3	NCBI accession: FJ411193; NM_174246.2; NCBI *Bos taurus* trace archive: gnl|ti|1078358515 nd others	Dr. J. Chae, University of Chonbuk, Korea
alpaca	*Lama pacos*	Ex1-Ex2-Ex3	NCBI *Lama pacos* trace archive: gnl|ti|2127575520 and others	-
moose	*Alces alces*	pEx2-pEx3	NCBI accession: FJ411187, FJ411188	K. Zierau, Berlin, Germany
Perissodactyla				
horse	*Equus caballus*	Ex1-Ex2-Ex3	NCBI *Equus caballus* trace archive: gnl|ti|1236303957 and others; NCBI accession: XM_001491813.2	-
Carnivora				
domestic cat	*Felis catus*	pEx2-Ex3	NCBI *Felis catus* trace archive: gnl|ti|687346034 and others	-

common name	species	cloned sequence	NCBI accession /source	source of genomic DNA
lion	*Panthera leo*	pEx2-pEx3	NCBI accession: FJ411235	Dr. C. Pitra, IZW, Berlin, Germany
dog	*Canis lupus familiaris*	Ex1-Ex2-Ex3	NCBI accession: FJ411194; NM_001003177.1, AJ238150.1; NCBI *Canis familiaris* trace archive: gnl|ti|322378527 and others	own source
Eurasian river otter	*Lutra lutra*	pEx2-pEx3	NCBI accession: FJ411216, FJ411217	Dr. C. Pitra, IZW, Berlin, Germany
Baikal seal	*Phoca sibirica*	pEx2-pEx3	NCBI accession: FJ411238	Dr. C. Pitra, IZW, Berlin, Germany
California sea lion	*Zalophus californianus*	pEx2-pEx3	NCBI accession: FJ411250, FJ411251	Dr. C. Pitra, IZW, Berlin, Germany
Insectivora				
European shrew	*Sorex araneus*	Ex1-Ex2-Ex3	NCBI *Sorex araneus* trace archive: gnl|ti|999156174 and others	-
middle-African hedgehog	*Atelerix albiventris*	Ex1-Ex2-Ex3	NCBI *Atelerix albiventris* trace archive: gnl|ti|1243676371 and others	-
Chiroptera				
large flying fox	*Pteropus vampyrus*	Ex1-Ex2-Ex3	NCBI *Pteropus vampyrus* trace archive: gnl|ti|1325436861 and others	-
greater horseshoe bat	*Rhinolophus ferrumequinum*	Ex1-Ex2-Ex3	NCBI *Rhinolophus ferrumequinum* trace archive: gnl|ti|749841267 and others	-
little brown bat	*Myotis lucifugus*	Ex1-Ex2-Ex3	NCBI *Myotis lucifugus* trace archive: gnl|ti|981681939 and others	-
Afrotheria				
small Madagascar hedgehog	*Echinops telfairi*	Ex1-Ex2-Ex3	NCBI *Echinops telfairi* trace archive: gnl|ti|1588170346 and others	-
cape rock hyrax	*Procavia capensis*	Ex2-Ex3	NCBI *Procavia capensis* trace archive: gnl|ti|2062418997 and others	-
short-eared elephant shrew	*Macroscelides proboscideus*	pEx2-pEx3	NCBI accession: FJ411219	Dipl.-Biol. Christian Kern, Tierpark Berlin, Berlin, Germany
Caribbean manatee	*Trichechus manatus*	pEx2-pEx3	NCBI accession: FJ411247, FJ411248	Dr. C. Pitra, IZW, Berlin, Germany
Asiatic elephant	*Elephas maximus*	pEx2-pEx3	NCBI accession: FJ411200	Dr. C. Pitra, IZW, Berlin, Germany
African savanna elephant	*Loxodonta africana*	Ex1-Ex2-Ex3	NCBI accession: FJ411215; NCBI *Loxodonta africana* trace archive: gnl|ti|2074290547 and others	Dr. C. Pitra, IZW, Berlin, Germany
Xenarthra				
nine-banded armadillo	*Dasypus novemcinctus*	Ex1-Ex2-Ex3	NCBI *Dasypus novemcinctus* trace archive: gnl|ti|1814033013 and others	-

common name	species	cloned sequence	NCBI accession /source	source of genomic DNA
**Metatheria**<br>***Didelphimorphia***				
North American opossum	*Didelphis virginiana*	pEx2-pEx3	NCBI accession: FJ411198, FJ411199	ATCC - cell collection, OK
gray short-tailed opossum	*Monodelphis domestica*	Ex2-Ex3	NCBI *Monodelphis domestica* trace archive: gnl|ti|368796670 and others	-
Diprotodontia				
long-nosed potoroo	*Potorous tridactylus*	pEx2-pEx3	NCBI accession: FJ411240	ATCC - cell collection, Pt K1
red kangaroo	*Macropus rufus*	pEx2-pEx3	NCBI accession: FJ411222	Prof. Dr. Mario Mörl, University of Leipzig, Leipzig, Germany
agile wallaby	*Macropus agilis*	pEx2-pEx3	NCBI accession: FJ411221	Prof. Dr. Mario Mörl, University of Leipzig, Leipzig, Germany
tammar wallaby	*Macropus eugenii*	pEx2, Ex3	NCBI *Macropus eugenii* trace archive: gnl|ti|1377251165 and others	-
**Protheria**<br>***Monotremata***				
platypus	*Ornithorhynchus anatinus*	Ex1-Ex2-Ex3	NCBI *Ornithorhynchus anatinus* trace archive: gnl|ti|687747165 and others; XM_001520222.1	-

Abbreviations: p, partial; Ex, Exon
