# Supplementary material for: Involvement of the V2 Vasopressin Receptor in Adaptation to Limited Water Supply
Source: PLoS One. 2009 May 18;4(5):e5573. doi: 10.1371/journal.pone.0005573 (PMC2680020; doi:10.1371/journal.pone.0005573)
Supplement: Table S2 — Primers used for V2 ortholog amplification and cloning. (0.01 MB PDF) [file pone.0005573.s004.pdf]

Table S2. Primers used for V2 ortholog amplification and cloning.

primer number	sequence (5’- 3’)	orien-tation	name
1	NGTGGCRCAGGAYTCNTCTTGRGG	as	deg-V2-AS1
2	GGAGCTGGCNGTGGCRCAGGAYTC	as	deg-V2-AS2
3	NGCRTAGATCCARGGGTTGGTRCA	as	deg-V2-AS3
4	AGCTGCACNAGGAAGAANGGNGCCCA	as	UA-V2-TM6-less-deg-AS
5	CTGAAGRANGCRTAGATSCARGGGTT	as	UA-V2-TM7-part-fugo-AS
6	AGCTGCACNAGRAARAANGGNGYCCA	as	UA-V2-TM6-more-deg-AS
7	CGRGACCCGCTGCTNGYCCAGG	s	deg-V2-S1-Teil1
8	CGRGACCCGCTGCTNGYCCGGG	s	deg-V2-S1-Teil2
9	TTYGTGGCYGTGGCCYTGAGCAA	s	deg-V2-S2
10	TTYGTGGCYGTGGCCYTKRGMAA	s	V2-S2-more-deg-part-frog
11	GTGAAGTACYTSCANRTSGTNGGNATG	s	UA-V2-TM3-more-deg-S
12	GTGAAGTACCTGCAGRTSGTNGGNATG	s	UA-V2-TM3-less-deg-S
13	AGCGTACCTAGGCTCGAAGG	as	cow AVPR2 AS INTRON2
14	AGCGTGACGTGGATGGTAG	s	cow AVPR2 S Exon2
15	CCGGGCAGAATAGACTCACT	s	cow AVPR2 S INTRON1
16	TGTAGGCGGTCAGCTGAGTT	as	cowAVPR2 AS-C-Terminus 3'UTR
17	ACCTGACAGGCAGCAATACC	as	cowAVPR2 AS-N-Terminus-Exon2
18	ATGGCCTTCGAGCCTAGGTA	s	cowAVPR2 S-C-Terminus Intron 2
19	GGAGGCACCACGGGAAGGGCCTCCCTTCGTGTTG	s	cow AVPR2 S Exon2-Exon3-Adapt
20	CAACACGAAGGGAGGCCCTTCCCGTGGTGCCTCC	as	cow AVPR2 AS Exon2-Exon3-Adapt
21	GAGCTCACAACCTCGTCTCC	s	cow AVPR2 S vor Ex1
22	GATCCARGGGTTGGTRMAGCTG	as	Beutel_V2_AS1
23	GATCCAAGGGTTGGTGAAGCTG	as	Beutel_V2_AS2
24	TTYGTGKSYGTRRCMYTGAGCAA	s	Beutel_V2_S1
25	GTTGGCCCAGGCTGAGGTG	s	Beutel_V2_S2
26	GCGGAATTCCCTAACGGGGCCTCTTCCTC	s	dogV2-gDNA-vorEx2_S
27	CGCGAATTCGCTCCTAGTCCCAGAGCCCC	as	dogV2-gDNA-AS
28	CGGAATTCGGTACCGCCACCATGTACCCCTACG ACGTCCCCGACTACGCC	s	HA-huV2-EcoRI-KpnI-S
29	GACTAGTGCGGCCGCGCCGAGCTAGGGCCGC	as	NotI-SpeI-V2-TM1-AS
30	GACTAGTTCACTTATCGTCATCGTCCTTATAATCCG ATGAAGTGTCCTTGGCCAGGGA	as	FLAG-V2-AS
31	ATAAGAATGCGGCCGCCGGGGCCRCTGGGCRCCC AT	s	NotI-V2-TM1-S
32	CATAGATCCAGGGGTTGGTRCAGCTG	as	PflMI-V2-TM7-AS
33	CACTTATCGTCATCGTCCTTATAGTCGGAGGGGGT GTCCTTGG	as	cow AVPR2 AS Exon3-Flag-Adapt
34	CGTCCCCGACTACGCCTTCATGGCATCCACCACCT CAGCTGTGCCCTGGCACCT	s	cowAVPR2-S-HA-Adaptor-Exon1-Exon2 Adapt
35	CGTCCCCGACTACGCCTTCATGGCATCCACCACCT	s	cow AVPR2 S HA-Ad.auf Ex1
36	ATAAGAATGCGGCCGCCGAGGCCGGTGGGCCCCT AT	s	NotI_Beutel_S
37	GTCCCCGACTACGCCCTCCTGGCATCCACCACCTC GGCTGTGCCCCGGACCCTCT	s	dogV2-HA-Adaptor_Ex1_aufEx2_S
38	CTTATCGTCATCGTCCTTATAGTCGGAGAAGTGTC CTTGGCCAGGAAGG	as	dogV2-gDNA-AS-FlagAdaptor-auf_Ex3
39	CGTAGATCCAGGGGTTGGTGCAGCTG	as	dogV2_PfmlI_AS
40	GGCGCGCCGCGGCCGCCGGGGTCGCTGGGC	s	dogV2_NotI_S
