# Supplementary material for: Involvement of the V2 Vasopressin Receptor in Adaptation to Limited Water Supply
Source: PLoS One. 2009 May 18;4(5):e5573. doi: 10.1371/journal.pone.0005573 (PMC2680020; doi:10.1371/journal.pone.0005573)
Supplement: Table S3 — Missense mutations found in human V2R. (0.01 MB PDF) [file pone.0005573.s005.pdf]

Table S3. Missense mutations found in human V2R.

AA change	phenoptye
T7S	benign
G12E	benign
A42V	benign
L43P	NDI
L44P	NDI
L44F	NDI
I46K	NDI
L53R	NDI
S54R	NDI
N55D	NDI
N55H	NDI
L59P	NDI
A61V	benign
L62P	NDI
R64W	benign
H80R	NDI
L81F	NDI
L83P	NDI
L83Q	NDI
A84D	NDI
D85N	NDI
V88L	NDI
V88M	NDI
Q92R	NDI
L94Q	NDI
P95L	NDI
A98P	NDI
W99R	NDI
R104C	NDI
F105V	NDI
R106C	NDI
G107E	NDI
G107R*	NDI
C112R	NDI
C112Y	NDI
R113W	NDI
G122R	NDI
G122D	NDI
M123K	NDI
S126F	NDI
S127F	NDI
Y128S	NDI
I130F	NDI
I130L	NDI
A132D	NDI

AA change	phenoptye
L135P	NDI
R137H	NDI
R137C	NSIAD
R137L	NSIAD
R139S	benign
C142W	NDI
R143G	NDI
R143P	NDI
A147V	NDI
A163P	NDI
W164S	NDI
A165D	NDI
S167L	NDI
S167T	NDI
P173S	NDI
Q174L	NDI
Q174R*	NDI
R181C	NDI
G185C	NDI
S187R	NDI
D191G	NDI
C192R	NDI
G201D	NDI
R202C	NDI
R203C	NDI
T204N	NDI
Y205C	NDI
Y205F	NDI
V206D	NDI
T207N	NDI
I209F	NDI
F214S	NDI
V215M	benign
P217T	NDI
L219P	NDI
L219R	NDI
R230P	NDI
R252W	benign
M272K	NDI
L274P	NDI
V277A	NDI
Y280C	NDI
L282P	NDI
A285P	NDI
P286L	NDI

AA change	phenoptye
P286R	NDI
P286S	NDI
F287L	NDI
L289P	NDI
V290G	NDI
L292P	NDI
A294P	NDI
L309P	NDI
L309R	NDI
S315R	NDI
N317K	NDI
S318T	benign
C319R	NDI
N321D	NDI
N321K	NDI
N321Y	NDI
P322H	NDI
P322S	NDI
P322L	NDI
W323R	NDI
W323S	NDI
S329R	NDI
G352D	benign

References are given in (Bichet 2008; Spanakis, Milord, and Gragnoli 2008).
* missense mutation discovered and functionally analyzed by our laboratory (unpublished data)
